# Genomic Landscape of Chinese Clear Cell Renal Cell Carcinoma Patients With Venous Tumor Thrombus Identifies Chromosome 9 and 14 Deletions and Related Immunosuppressive Microenvironment

**DOI:** 10.3389/fonc.2021.646338

**Published:** 2021-06-23

**Authors:** Shaoxi Niu, Kan Liu, Yong Xu, Cheng Peng, Yao Yu, Qingbo Huang, Shengpan Wu, Bo Cui, Yan Huang, Xin Ma, Xu Zhang, Baojun Wang

**Affiliations:** ^1^ Department of Urology, The Third Medical Centre, Chinese PLA (People’s Liberation Army) General Hospital, Beijing, China; ^2^ Department of Urology, The Seventh Medical Center of Chinese PLA General Hospital, Beijing, China

**Keywords:** venous tumor thrombus, ccRCC, genomic feature, copy number variant, immune microenvironment

## Abstract

**Background:**

Clear cell renal cell carcinoma (ccRCC) with venous tumor thrombus (VTT) is associated with a poor clinical outcome. Although several studies have examined the genomic features of ccRCC, the genetic profile of VTT along with its matched primary tumor has not been fully elucidated.

**Materials and methods:**

Samples of VTT tissues and matched primary tumor tissues from ccRCC patients (n = 25), as well as primary tumor tissues from patients without VTT (n = 25) were collected and analyzed using whole-exome sequencing. Four additional ccRCC patients who were unfit for surgery were treated with an anti-programmed death receptor-1 (PD-1) monoclonal antibody (Toripalimab, 240 mg, Q3W, IV).

**Results:**

By comparing the primary kidney tumors from ccRCC patients with or without VTT, a relatively higher prevalence of *BAP1 and KDM5C* alterations were found in ccRCC patients with VTT, and these alterations were associated with worse overall survival in the kidney renal clear cell carcinoma (KIRC) database. Based on subclone analysis, VTT was predicted to primarily originate directly from the primary renal mass. A significantly higher prevalence of *CELSR2* and *TET2* alterations were identified in the VTTs compared with the matched primary tumors. An increased prevalence of DNA damage repair genes, especially those involved in homologous recombination repair and non-homologous end joining, was found in ccRCC patients with VTT. Notably, VTT was characterized by the increase incidence of copy number loss in the whole exome (*p* < 0.05), particularly in the chromosome 9 and 14 regions. Deletion of chromosome 9 and 14 was associated with worse survival, unfavorable clinical features, and the presence of an immunosuppressive microenvironment, which was characterized by higher infiltration of regulatory T cells, follicular helper T cells, and resting mast cells, but lower counts of resting CD4 memory T cells and CD8 positive T cells. A significantly lower count of CD4+ and CD8+ tumor-infiltrated lymphocytes was identified in the VTT samples comparing with matched primary tumor. Of note, three out of the four ccRCC patients with VTT in our cohort who were treated with the anti-PD-1 therapy exhibited remarkable remission in the renal mass but no notable shrinkage in the VTT mass.

**Conclusion:**

Our study revealed the genetic profile of Chinese ccRCC patients with VTT, and identified multiple features associated with known poor outcomes, including gene alterations and copy number loss. The deletions in chromosomes 9 and 14, and the associated immunosuppressive microenvironment may indicate limited sensitivity to anti-PD-1/PD-L1 monotherapy in VTT.

## Introduction

Renal cell carcinoma (RCC) is the second most common genitourinary malignancy in China, with an estimated 66,800 new cases and 23,400 deaths in 2015 (1). Notably, 4–10% of locally advanced RCC patients develop venous tumor thrombus (VTT), and their overall 5-year cancer-specific survival (CSS) is only 40–65% (2). The median survival of untreated RCC patients with VTT is only 5 months, and the 1-year survival rate is less than 30% (3). To date, radical nephrectomy with thrombectomy remains the best therapeutic choice for RCC patients with VTT, and notably, a higher VTT level under the Mayo classification system is associated with worse CSS, increased rates of complications, and increased mortality following surgery (4, 5). To improve the safety and therapeutic effects of surgery, neoadjuvant therapies have been developed to shrink both the primary lesion and the VTT. Although previous studies have reported the use of anti-angiogenesis drugs preoperatively, including sorafenib and sunitinib, the clinical benefits are limited (6). Therefore, it is essential to understand the molecular mechanisms underlying the formation and development of VTT.

A few previous studies have reported the genomic features of RCC with VTT. As depicted by the TRACERx Renal project, most genetic alterations in the VTT tissues were also present in their matched primary tumor tissue; however, the primary tumor tissues also possessed more recently developed driver mutations that were absent from the VTT tissue (7). Similarly, another study also suggested that the VTT originated from the primary tumor, as all subclones in the VTT were also shown to match the primary renal tumors (8). In the same study, a homologous recombination deficiency feature, described as the BRCAness mutation signature, was also identified in a subset of RCC tumors with VTT and samples from The Cancer Genome Atlas (TCGA), indicating a DNA damage repair (DDR) deficiency and potential sensitivity to poly(ADP-ribose) polymerase inhibitors or platinum-based therapy in these patients (8). Nevertheless, most research on the genomic landscape of RCC was performed in Caucasians, and the results may differ for Chinese RCC patients with VTT, particularly considering the different genetic backgrounds and the exposure to aristolochic acid in traditional Chinese medicines.

In this study, we applied whole-exome sequencing to study the genomic features of VTT in Chinese patients with clear cell (cc)RCC with the matched primary tumors, and these were also compared with primary tumors from RCC patients without VTT to elucidate the genomic features and their potential clinical significance in ccRCC with VTT.

## Material and Method

### Sample Source and Ethic Data

We prospectively enrolled 50 ccRCC patients at the Third Medical Centre of Chinese PLA (People’s Liberation Army) General Hospital from 2018 to 2019. Twenty-five were ccRCC with VTT, and twenty-five were ccRCC without VTT. All patients had undergone radical nephrectomy with or without thrombectomy and provided written informed consent. This study was approved by the ethics committee of the First Medical Center of PLA General Hospital (S2017-100-01) and conducted under the principles of the Declaration of Helsinki and the Good Clinical Practice guidelines. Patient characteristics were listed in **Table 1**. All samples were collected for DNA isolation after pathological evaluation, but two VTT samples did not yield enough DNA for further testing. Finally, 23 VTT samples (V group), 25 primary tumor samples (VP group), and 25 renal tumor samples without VTT (NP group) were analyzed by whole-exome sequencing (WES), using the DNA from peripheral blood as germline control. In addition, another four ccRCC patients with VTT were evaluated as unfit for surgery and were treated with anti-programmed death receptor-1 (PD-1) monoclonal antibody (Toripalimab, 240 mg, Q3W, IV).

**Table 1:** Clinical Characteristics of 50 RCC Patients.

Characteristics		With VTT n = 25	Non-VTT n = 25
Median age, year (range)		52 (25–84)	55 (34–86)
Sex			
	Male	21	20
	Female	4	5
ISPU Grade			
	1	0	1
	1-2	0	1
	2	12	12
	2-3	6	7
	3	5	3
	3-4	2	1
	4	0	0
Histological			
subtype	Clear cell RCC	25	25
			
IVC wall	Yes	19	–
invasion	No	6	–

### DNA Isolation

DNA was extracted using DNeasy Blood & Tissue Kit (Qiagen, Inc.) under the manufacturer’s instructions. The purified gDNA was quantified using Qubit 3.0 Fluorometer (Life Technologies, Inc.) and StepOnePlus System (Life Technologies, Inc.). For tumor and non-tumor samples, 100 ng of DNA was sheared with a Covaris E210 system (Covaris, Inc.) to generate fragments with a length of 200 bp.

### WES

The Accel-NGS 2S HYB DNA LIBRARY KIT (Swift Biosciences, 23096) and HotStart ReadyMix (KAPA, KK2612) for library preparation and amplification were used, respectively. The amplified libraries were purified by using SPRISELECT (Beckman, B23319) and further captured with xGen Exome Research Panel v2 (IDT), whose target region was 33 Mb. Finally, samples underwent paired-end sequencing on a Novaseq 6000 platform (Illumina) with a 150 bp read length. The mean depth for the tumor, VTT and non-tumor tissues was 500×, 500×, and 100×, respectively.

### Data Analysis

Raw sequencing data were aligned to the reference human genome (UCSC hg19) through Burrows-Wheeler Aligner (9). After deduplication and local realignments, Genome Analysis Toolkit (GATK) was used for calling of single nucleotide variation (SNV) and small insertion and deletion (indel) (10). Somatic variants that were present only in tumor or VTT were identified by removing the germline alterations identified in the matched non-tumor samples. Variants were annotated by using the ANNOVAR software (11). CNVkit was used to determine the copy number variations (CNVs) (https://github.com/etal/cnvkit).

### Tumor Mutation Burden

The tumor mutation burden of each sample was calculated according to the widely used method described by Chalmers, Z.R, et al. (12).

### Mutational Signatures

Mutational signatures were analyzed by R package YAPSA with supervision. A linear combination decomposition of the mutational catalog with known and predefined signatures was computed by non-negative least squares (NNLS). By comparing the whole genome to WES capture regions, mutational catalog correction was performed to account for the differences in the occurrence of triplet motifs. A set of 30 publicly available mutational signatures AC1-AC30 (AC standing for Alexandrov COSMIC) were analyzed.

### Homologous Recombination Deficiency

The homologous recombination deficiency, also called genomic scar scores was determined by counting the number of loss of heterozygosity (LOH), large scale transitions (LSTs), and telomeric allelic imbalances (TAIs). WES sequencing data analysis was performed by using the method described by Zsofia Sztupinszki et al. (13), which showed a good correlation (r = 0.87) between SNP array-based and WES sequencing-based HRD analysis.

### Gene Expression Signature Analysis

Gene expression signature of kidney renal clear cell carcinoma (KIRC) in TCGA was analyzed based on the RNA-seq data (downloaded for cbioportal, https://www.cbioportal.org). Signatures were classified into angiogenesis, immune and antigen presentation, myeloid inflammation according to the IMmotion 150 trial (14).

### Tumor-Infiltrating Immune Cell Analysis

Tumor-infiltrating immune cell counts were analyzed based on RNA-seq data from the KIRC in TCGA by using a CIBERSORT R package (15).

### Immunohistochemical Detection of CD3, CD4, and CD8

Immunohistochemistry (IHC) analysis on the archived tumor samples was applied to compare the expression level of CD3, CD4, and CD8 in tumor infiltrated lymphocytes. Anti-CD8 (EP1150Y, ab93278), anti-CD4 (EPR6855, ab133616), and anti-CD3 (SP7, ab16669) antibody were purchased from Abcam (Cambridge, MA). All experiments were undergone following the instructor’s protocol. Two pathologists independently interpreted IHC staining by assessing background staining, positive and negative controls, and localization and amount of biomarker staining in all specimens. Percent positive cells = (number of positive lymphocytes/tumor area occupied by tumor cells, associated intratumoral, and contiguous peritumoral stroma) × 100.

### Statistical Analysis

Gene prevalence between different groups was analyzed by Chi-Square test or Fisher exact test. A two-sided P value of less than 0.05 was considered to be statistically significant. All analyses were performed using SPSS 25.0 software.

## Results

### Genomic Landscape of the ccRCC Patients in our Cohort

**Figure 1:**
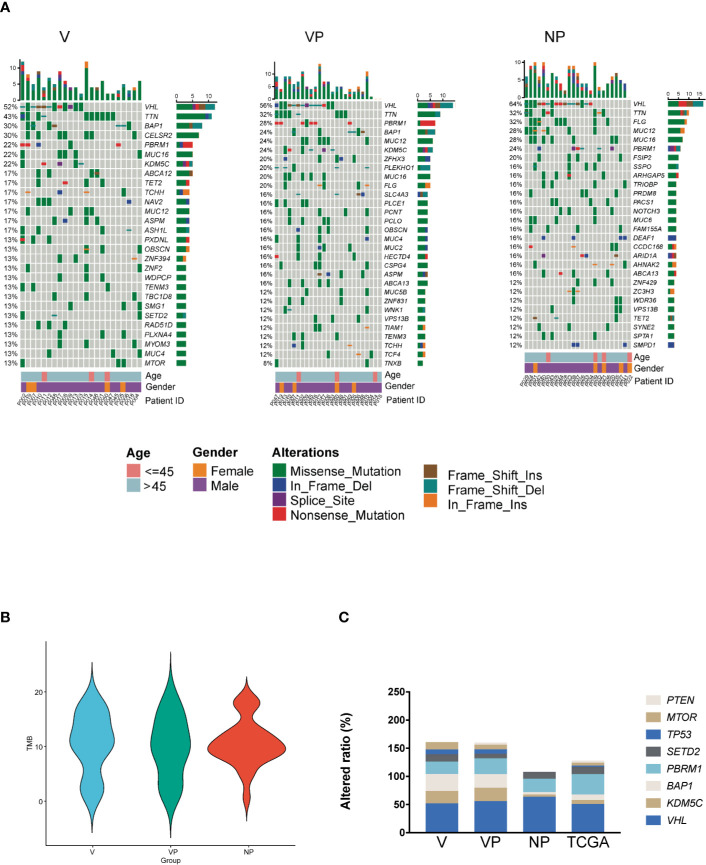
Somatic mutation landscape of ccRCC with and without VTT. **(A)** Mutation landscape of the NP (n = 25), VP (n = 25), and V (n = 23) samples. The vertical histogram shows the number of different mutational types in each sample. The heat map shows the distribution of top genes across samples. The horizontal histogram shows the number of different mutational types in each gene. **(B)** The tumor mutation burden of the V, VP, and NP groups. **(C)** The frequency of mutations in driver genes of ccRCC in the three groups (V, VP, and NP) and in data obtained from TCGA. TCGA, The Cancer Genome Atlas; VTT, venous tumor thrombus; ccRCC, clear cell renal cell carcinoma; V, VTT; VP, matched primary tumor; NP, normal primary tumors without VTT.

Overall, we identified 9,879 non-synonymous somatic alterations in the 73 samples. The mean and median numbers of somatic alterations per sample were 135.33 and 107, respectively. The most frequently altered gene was *VHL*, with an approximately 60% alteration rate in all three groups. In the V group, the other frequently altered genes included *TTN* (43%), *BAP1* (30%), *CELSR2* (30%), *PBRM1* (22%), *KDM5C* (22%), and *MUC16* (22%), respectively (**Figure 1A**). All samples in the three groups were microsatellite instability stable (MSS), and there was no significant difference in the median tumor mutation burden (**Figure 1B**).

To identify the different genomic features between Chinese and Western cohorts, we compared the prevalence of alterations in known driver genes in ccRCC, including *VHL*, *KDM5C*, *BAP1*, *PBRM1*, *SETD2*, *MTOR, TP53*, and *PTEN* between our cohort and KIRC database from TCGA. The prevalence of *VHL* alterations was similar between our cohort and data obtained from TCGA, whereas the prevalence of *KDM5C*, *BAP1*, *TP53*, and *PTEN* alterations were different (**Figure 1C**, p > 0.05). Notably, a high prevalence of *KDM5C* and *BAP1 alterations* was identified in both the primary tumor and VTT tissues from ccRCC patients with VTT in our cohort (*KDM5C*: 21.74, 24.00, 4.00, and 7.00%; *BAP1* 30.43, 24.00, 4.00, and 10.00% for V, VP, NP, and TCGA, respectively). No pathogenic or likely pathogenic germline variants in ccRCC-related susceptible genes were identified in all three groups of our cohort.

### Comparison of the Somatic Alterations Between ccRCC With or Without VTT

**Figure 2:**
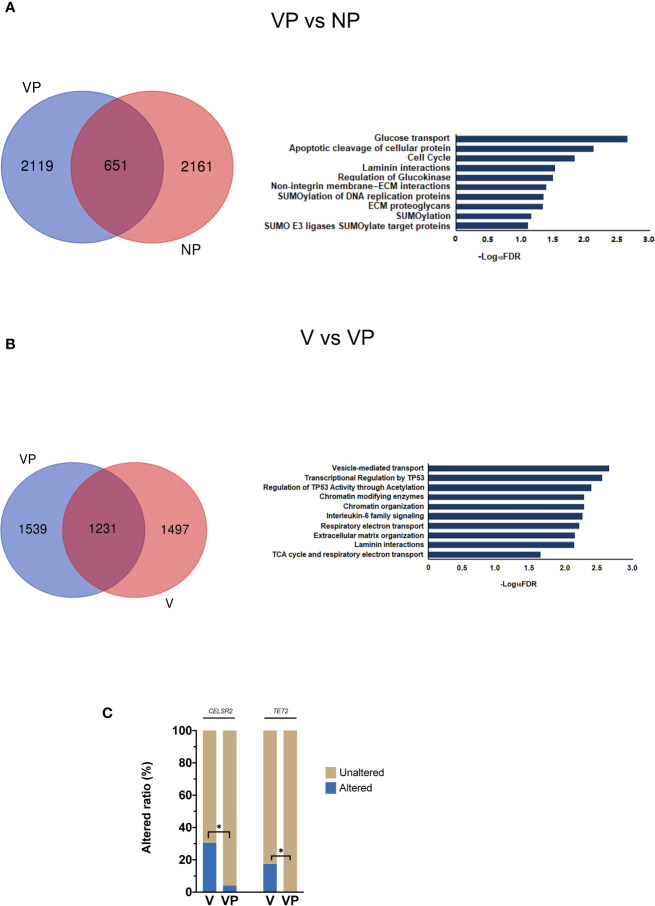
Analysis of differences in mutant gene prevalence. **(A)** Venn diagram and reactome pathway analysis of the differentially mutated genes in the VP and NP groups. **(B)** Venn diagram and reactome pathway analysis of the differentially mutated genes in the V and VP groups. **(C)** Differences in *CELSR2* and *TET2* mutational prevalence between the V and VP groups. *p < 0.05. VTT, venous tumor thrombus; V, VTT; VP, matched primary tumor; NP, normal primary tumors without VTT.

First, we compared the prevalence of altered genes in the primary tumors (VP *vs*. NP). Of the 4,931 mutated genes, only 651 genes were shared by the two groups (**Figure 2A**). The majority of the mutated genes had a similar prevalence in those two groups, whereas the genes with a different altered frequency were mainly involved in the following pathways: glucose transport, apoptotic cleavage of cellular protein, cell cycle, laminin interactions, and regulation of glucokinase. No gene with a significantly higher prevalence was found in the VP group.

### Comparison of the Somatic Alterations Between VTT and Matched Primary Tumors

Next, we compared the altered genes between VTT tissues and matched primary tumors (V *vs*. VP). Compared with the NP group, more genes were co-mutated in the V and VP groups. The genes with a different prevalence in VTT were primarily involved in vesicle-mediated transport, interleukin-6 (IL-6) family, transcriptional regulation by *TP53*, and the regulation of p53 activity through acetylation and Chromatin modifying enzymes (**Figure 2B**). We also identified two genes with significantly higher prevalence in the VTT tissues compared with the matched primary tumor tissues, including *CELSR2* (30.43 *vs*. 4.00%, *p* < 0.05) and *TET2* (17.39 *vs*. 0, *p* < 0.05, **Figure 2C**). Interestingly, the prevalence of *CELSR2* was also higher in the V group compared with the NP group (30.43 *vs*. 4.00%, *p* < 0.05).

### Analysis of the Subclone Phylogeny Between the VTT and Matched Primary Tumors

**Figure 3:**
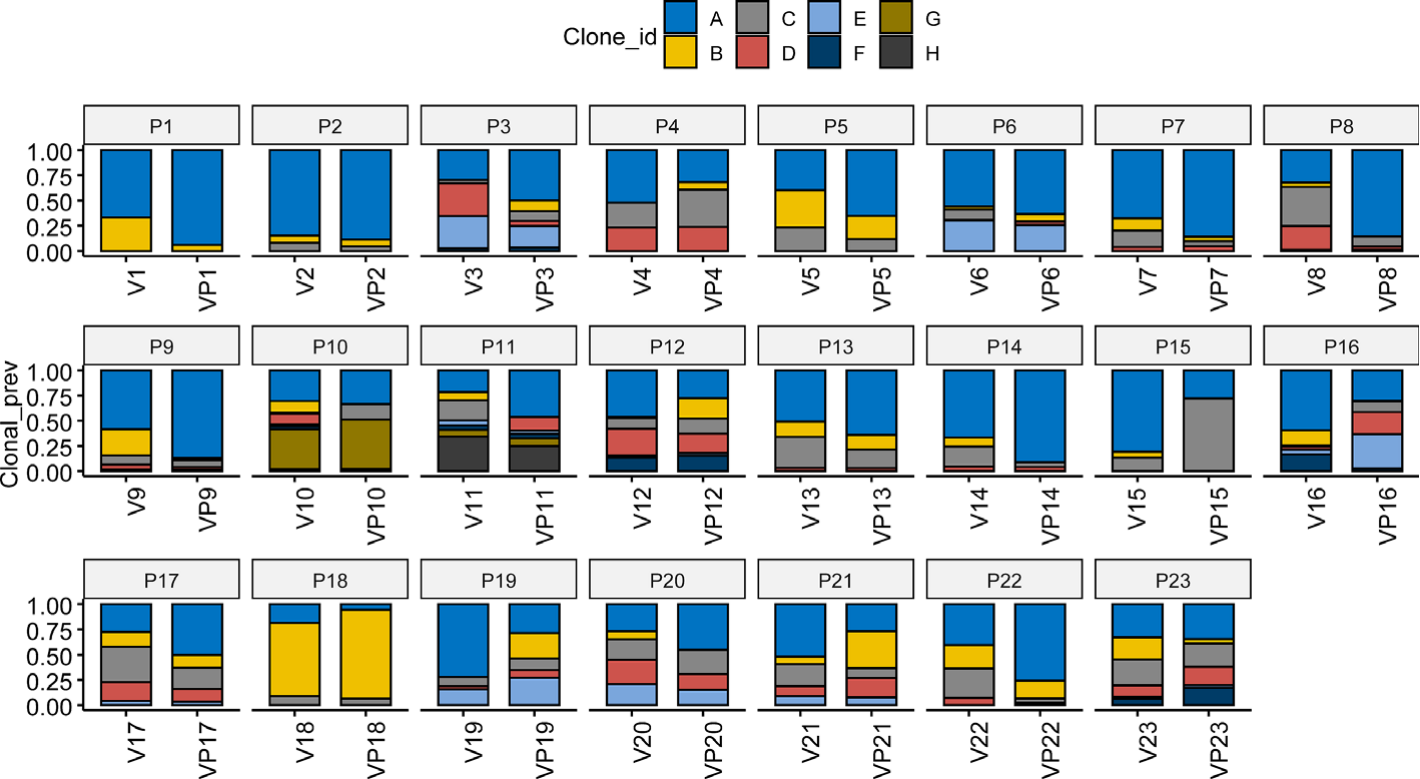
Analysis of clonal phylogeny between the VTT and matched primary tumor. The constitution of subclones in each VTT and its matched primary tumor. V, VTT; VP, matched primary tumor.

Pyclone was used to reconstruct the clonal population for each VTT and its matched primary tumor sample (16). As shown in **Figure 3**, the bars with distinct colors represented different subclones in paired samples. The majority (15/23) of the paired samples shared the same subclones. However, eight VTT samples had notably distinct subclones from their matched primary tumors, suggesting the existence of genomic heterogeneity between the VTT tissue and its primary tumor.

### DNA Damage Repair Gene Alterations and Related Signatures

**Figure 4:**
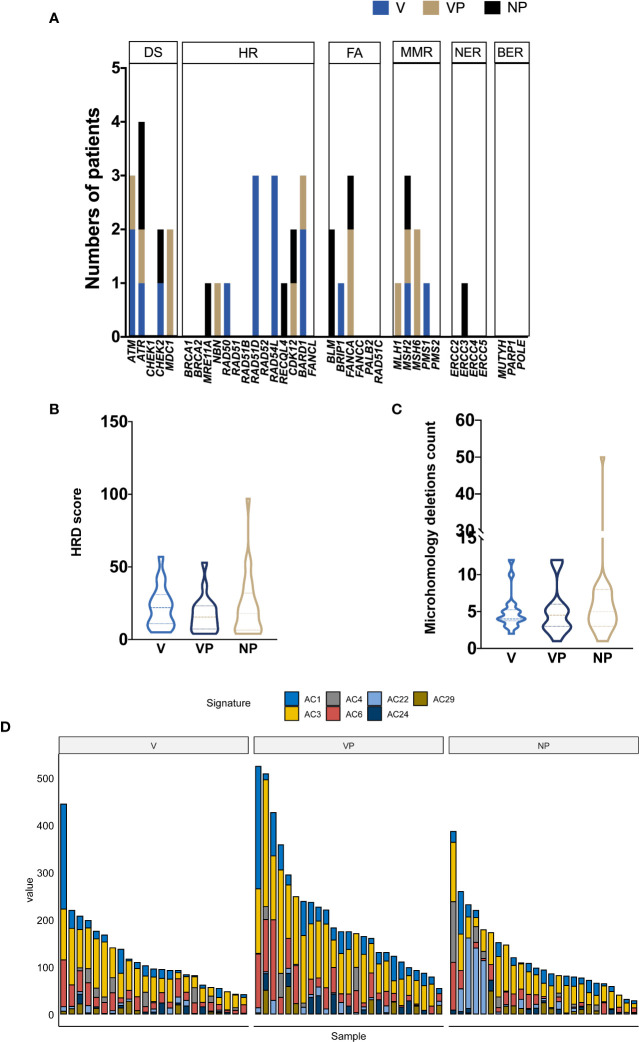
Mutational signature profile and DNA damage repair pathway analysis. **(A)** The distribution of DDR somatic mutations in each group (V, VP, and NP). The prevalence of HRD score **(B)** and microhomology deletions **(C)**. **(D)** Mutational signature in each group (NP, V, and VP). AC, Alexandrov COSMIC; DS, damage sensor; HR, homologous recombination repair; DDR, DNA damage repair; FA, Fanconi anemia; MMR, mismatch repair; NER, nucleotide excision repair; BER, base excision repair; VTT, venous tumor thrombus; V, VTT; VP, matched primary tumor; NP, normal primary tumors without VTT; HRD, homology recombination deficiency.

Given the potential association between the DNA damage repair (DDR) pathway and VTT reported by previous studies, we investigated the prevalence of gene alterations involved in specific DDR pathways (**Figure 4A**). The most frequently mutated DDR pathway was the homologous recombination repair (HR) pathway in all three groups, whereas no alteration in the base excision repair (BER) genes was identified. A trend of increase prevalence of alterations in DDR genes, especially homologous recombination repair and non-homologous end joining genes, was found in both the V and VP groups. The median homology recombination deficiency scores of V, VP, and NP groups that were 22.5, 15.5, and 19, respectively, did not differ significantly between the groups (**Figure 4B**). Similarly, no difference in the microhomology deletions, a signature of microhomology-mediated end-joining, was found among the three groups (**Figure 4C**). Next, we analyzed the mutation signature in these groups. Known signatures, including AC1 (related to spontaneous deamination), AC3 (associated with defects in DNA double-strand break repair by homologous recombination), AC4 (associated with smoking), AC6 (associated with defective DNA mismatch repair), AC22 (resulting from exposure to aristolochic acid), AC24 (resulting from exposures to aflatoxin), and AC29 (related to habit of chewing tobacco), were identified in all three groups (**Figure 4D**). Although a trend of a higher portion of AC3 was found in V and VP groups (*versus* NP group), it did not reach statistical significance.

### Copy Number Variation in ccRCC With and Without VTT

**Figure 5:**
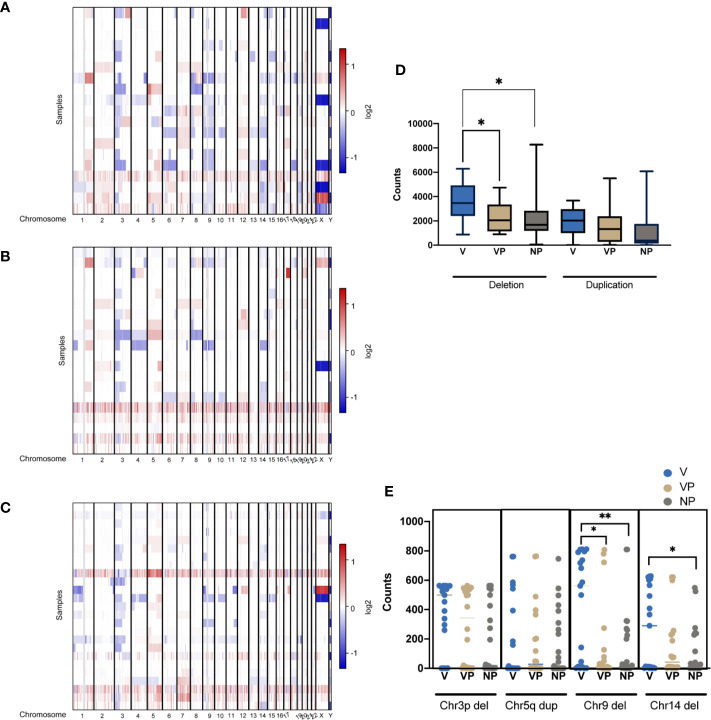
CNVs of tumors were plotted by chromosomal location (vertical axis) of the entire dataset in the V **(A)**, VP **(B)**, and NP groups **(C)**. **(D)** Differences in the copy number deletions and duplications in the three groups. **(E)** The counts of deletion of chromosome 3p (Chr3p Del), duplication of chromosome 5q (Chr5q Dup), deletions of chromosome 9 (Chr9 Del) and chromosome 14 in the three groups. *p < 0.05; **p < 0.01. CNV, copy number variation; VTT, venous tumor thrombus; V, VTT; VP, VTT matched primary tumor; NP, normal primary tumors without VTT.

CNV features were analyzed in all three groups (**Figures 5A–C**). Notably, the VTT samples had significantly more deletions compared with the VP (*p* = 0.011) and NP (*p* = 0.013) groups (**Figure 5D**). By contrast, no differences were found in the levels of copy number gain among the three groups. We also identified a significantly higher prevalence of chromosome 9 deletion in the VTT samples (**Figure 5E**). Compared with the NP group, a significantly higher prevalence of chromosome 14 deletion was found in the V group (**Figure 5E**).

### Clinical Features and the Tumor Immune Microenvironment in ccRCC Patients With Chromosome 9 and/or 14 Deletion

**Figure 6:**
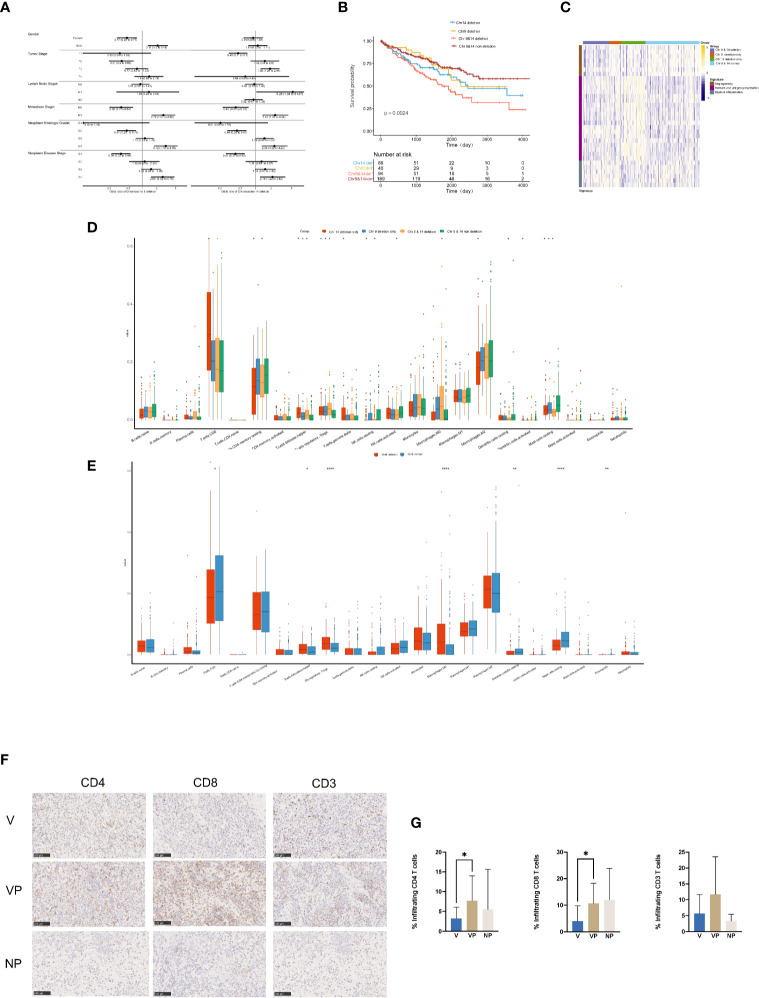
Clinical features and tumor microenvironment of the ccRCC patients with deletions in chromosomes 9 and 14 based on the KIRC database. **(A)** Clinical features of the ccRCC patients in the KIRC database with or without deletions in chromosome 9 (left) or 14 (right). **(B)** Kaplan–Meier curves for OS of ccRCC patients in the KIRC database with or without chromosome 9 and/or 14 deletions. **(C)** Heat map of gene expression signatures in angiogenesis, immune and antigen presentation and myeloid inflammation of ccRCC patients in the KIRC database with or without chromosome 9 and/or 14 deletion. **(D)** Analysis of tumor-infiltrating immune cell types in ccRCC patients in the KIRC database with or without chromosome 9 and/or 14 deletion. **(E)** Analysis of tumor-infiltrating immune cell types in ccRCC patients in the KIRC database with or without deletions in chromosome 9. Immunostaining **(F)** and analysis **(G)** for CD4, CD8 and CD3 in the V, VP, and NP groups. **(G)** *p < 0.05; **p < 0.01; ***p < 0.001. ccRCC, clear cell renal cell carcinoma; KIRC, kidney renal clear cell carcinoma; OS, overall survival; V, VTT; VP, VTT matched primary tumor; NP, normal primary tumors without VTT.

To investigate the clinical features in ccRCC patients with chromosome 9 and/or 14 deletion, we analyzed the clinical data and tumor immune microenvironment in the KIRC database. In total, 32.68% (134/410) and 43.90% (180/410) of the ccRCC patients in KIRC had chromosome 9 or 14 deletion, respectively, which was associated with an increased incidence of distant metastasis (26.1 *vs* 11.2% and 22.8 *vs* 10.9%, respectively, p < 0.01) and neoplasm disease stage of stage 4 (26.3 *vs* 11.6% and 22.2 *vs* 11.7%, respectively, p < 0.01) in comparison with the cases with neither chromosome 9 nor chromosome 14 deletions (**Figure 6A**). In agreement with the unfavorable clinical features, chromosome 9 and chromosome 14 deletions were also significantly associated with worse overall survival, particularly for patients with both chromosome 9 and chromosome 14 deletions (**Figure 6B**).

The heat map of the expression levels of genes involved in angiogenesis, immune and antigen presentation, and myeloid inflammation was shown in **Figure 6C**. There was a relatively lower expression of genes associated with angiogenesis in samples with chromosome 4 deletion, but a higher expression of genes involved in myeloid inflammation in ccRCC patients with chromosome 9 deletion in the KIRC database. Furthermore, there was a significantly higher accumulation of follicular helper T cells, regulatory T cells (Tregs) and macrophage M0 cells, but a significantly lower number of CD4 memory resting cells, resting mast cells were identified in ccRCC patients with chr9 and/or chr14 deletion in KIRC (**Figure 6D**). Furthermore, a significantly lower content of CD8 positive cells was identified in ccRCC patients with chromosome 9 deletion in KIRC (**Figure 6E**). Notably, by immunochemistry analysis, there was a significantly lower count of CD4+ and CD8+ tumor-infiltrated lymphocytes in the VTT samples compared with their primary tumor tissues from our cohort (**Figures 6F, G** **)**.

### The Response to Immune Checkpoint Inhibitors Differs Between VTT Tissues and the Renal Mass

**Figure 7:**
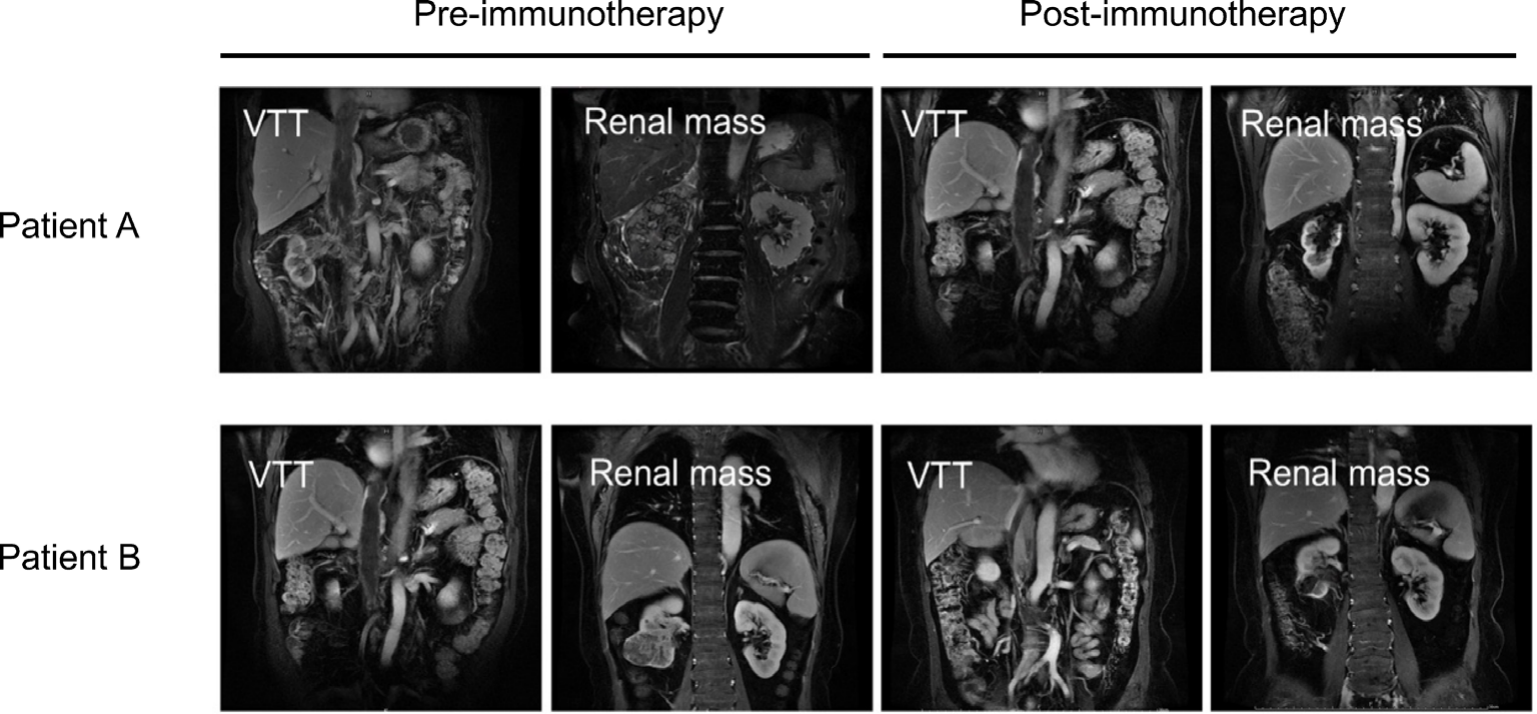
Images of VTT and the renal mass pre- (left) or post-immunotherapy (right) of two ccRCC patients with VTT. VTT, venous tumor thrombus; ccRCC, clear cell renal cell carcinoma.

A total of four ccRCC patients with VTT in our center who were unfit for surgery in the initial evaluation were treated with anti-programmed death receptor-1 (PD-1) monoclonal antibody treatment (Toripalimab, 240 mg Q3W IV) (**Supplementary Table 1**). One patient stopped treatment after only three cycles of Toripalimab due to personal reasons. The other three finished the six cycles of therapy, and all three exhibited significant reduction of the renal mass after six cycles of immune checkpoint inhibitor (ICI) treatment. However, the VTT did not regress in two of the three ccRCC patients with VTT (**Figure 7**). Patient A’s renal mass decreased from 9.56 to 4.31 cm in diameter after immunotherapy; however, his thrombus did not show significant regression (19.31 to 18.52 cm in diameter); Patient B also had a notable response in the renal mass (6.45 to 2.84 cm), but not in the VTT lesion (13.14 to 13.03 cm).

## Discussion

The 5-year survival rate of RCC has been strikingly improved to 74% over the past decades, and this was mainly attributed to the successful advances of targeted drugs and ICI (17). However, for ccRCC patients with VTT, the efficacy of neoadjuvant and adjuvant treatments remains contested (18). A comprehensive understanding of the genomic feature of VTT and its differences from the primary tumor may assist clinicians with regard to therapeutic decisions.

Consistent with the genetic profile of ccRCC patients in the KIRC and literature, the most common genetic alterations we identified in our patients, either with or without VTT, were *VHL*, *PBRM1*, *BAP1*, and *KDM5C*, which are all known driver genes in ccRCC (19). Compared with ccRCC patients without VTT and the corresponding data in the KIRC, we found a trend of an increased prevalence of *BAP1* alterations in both VTT and their matched primary tumor tissues, and alterations of *BAP1* have been widely regarded as an unfavorable prognosis biomarker in ccRCC (20). Limited by the sample sizes, the prevalence of the majority of mutated genes did not differ significantly between the primary tumor tissues from ccRCC with or without VTT.

As for the genetic difference between VTT and the respective primary tumor, no studies have reported a difference in the prevalence of mutated genes, to the best of our knowledge. In this study, we found a significantly higher incidence of *CELSR2* and *TET2* alterations in the VTT compared with the matched primary tumors. *CELSR2*, which encodes a family member of the cadherin EGF LAG seven-pass G-type receptors, has been identified in 1.8% of ccRCC patients in the KIRC database, which is not statistically different from the frequency in our cohort (4.00% in the VP group). Notably, *CELSR2* was observed in 30.43% of the V group. Though it has been found to be involved in the contact-mediated intercellular communication and was suggested to participate in kidney development and physiology, the specific function of *CELSR2* in the ccRCC has not been clarified (21). *TET2* encodes a methylcytosine dioxygenase that was shown to be involved in DNA demethylation, and it is frequently mutated in myeloid malignancies and other disorders (22). *TET2* activation, which can be induced by Ascorbic acid, may lead to the loss of hydroxymethylcytosine, which is associated with a more adverse prognosis in ccRCC (23). The presence of frequent *CELSR2* and TET2 mutation in VTT suggested that these genes may be involved in the metastasis of the ccRCC cells, or alternatively, they may contribute to cell survival and/or proliferation in the thrombus, which is a novel environment distinct from the kidney. Further studies are required to reveal the cellular and molecular functions of these genes.

A previous study by Gregor Warsow et al. reported the presence of signature AC3 in VTT, an indicator for homology recombination deficiency (8). However, in our cohort, AC3 was not significantly more prevalent in the VTT tissues (**Figure 4D**). The difference in the results may be due to the widely spread genomic instability in our ccRCC cohort, regardless of the presence of VTT or not. A retrospective study in MSKCC found that 17% of metastatic ccRCC patients possessed alterations in DDR genes, and this was associated with better overall survival in immunotherapy-treated cohort, but not in the tyrosine kinase inhibitor-treated cohort (24). A previous study also found an unfavorable prognostic role of homologous recombination deficiency in ccRCC based on the data from TCGA (25). In the present study, a relatively higher incidence of DDR gene alterations was found in VTT and their matched primary samples compared with the NP group, suggesting the presence of genomic instability and metastasis-prone features in VTT and its primary tumor.

Notably, VTT samples possessed an increased number of CNV alterations; in particular, a higher number of CNV loss than the respective primary lesions, as well as when compared with ccRCC without VTT. We found a significantly higher prevalence of deletions in chromosome 9 and other chromosomes in the VTT group, which contained multiple tumor suppressor genes (including *PTPRD*, *CDKN2A*, *CDKN2B*, *BNC2*, *FANCC*, *TGFBR1*, *TLE4*, *TLE1*, *TSC1*, *PTCH1*, *KLF4*, *ROBO1*, *RAD51B*, and *MAX*), suggesting that the deletion of these tumor suppressor genes may contribute to either the metastasis and or thrombosis process. Deletions in the chromosome 9 have been found to be associated with a more aggressive clinicopathological feature in ccRCC, including more advanced stage diseases, larger tumor volume and notably, increased renal vein invasion, and may thus serve as an independent predictor of recurrence and survival following surgery (26). Additionally, deletions of regions in chromosome 9 were predictive of a worse prognosis based on the KIRC database. Moreover, our study identified a higher prevalence of chromosome 14 deletion in the VTT tissues, which contained a pivotal regulator in ccRCC, hypoxia-inducible factor 1*α* (HIF1*α*) (27). Previous studies have demonstrated that chromosome 14 deletion is associated with lower HIF1*α* levels and poor prognosis in ccRCC patients (28). It may be of value for examining the role of t chromosome 9 loss, as well as HIF1*α* deletion in the formation and development of VTT in future.

In addition to the tumor suppressor genes, we also found a unique tumor microenvironment feature in ccRCC patients with chromosome 9 deletions. Tumor-infiltrating immune cells in the microenvironment serve an essential function in tumor development, metastasis, and response to ICIs (29, 30). Recently, biomarker analysis of the JAVELIN Renal 101 trial showed that ccRCC patients with different gene expression signatures of immune and angiogenesis functions possessed distinct responses to avelumab plus axitinib or axtinib monotherapy (31). Interestingly, we found both a relatively lower accumulation of the angiogenesis signature and a higher accumulation of the myeloid inflammation signature in ccRCC patients with chromosome 9 deletions in the KIRC database, which may be related to the lower response rate to either the anti-angiogenesis or ICI monotherapy in ccRCC patients. The potential immunosuppressive feature of ccRCC with chromosome 9 deletions was further shown by the analysis of immune cell features, which showed a notably lower count of CD8 positive T cells, but higher levels of Treg cells. Treg cells act as a negative regulator of anti-tumor immunity by inhibiting the activation and differentiation of CD4 and CD8 positive T cells (32). Additionally, a higher level of tumor associated macrophages and/or a reduced level of CD8 positive T cells has also been correlated with a lower response rates to ICI monotherapy (33). Notably, for the three patients who received ICI therapy, ICI had potent beneficial effects on the primary renal mass, but not on the VTT, which may be due to the differences in the immune microenvironments present between VTT and the primary kidney lesions. Unfortunately, these patients were unfit for surgery, and we could not obtain the primary tumor and VTT tissues for genetic testing, which precluded the direct analysis of the correlation between the efficacy of immunotherapy and the genomic features. Nevertheless, this finding may serve as a clue for further clinical research on the immune microenvironment of VTT and immunotherapy.

In summary, a unique genomic feature, including chromosome 9 and 14 deletions was identified in this study, which may be associated with the development and/or maintenance of VTT. The deletions of chromosome 9 and 14 (particularly chromosome 9) may be associated with a suppressive immune microenvironment, suggesting a poor response to ICI monotherapy in the VTT of the ccRCC patients.

## Limitations

This study was limited by the sample size to draw any conclusions and vulnerable to selection bias. Future studies with larger cohorts are required to validate the clinical implications of the genomic features identified by the current study, as well as to reveal additional features that are only attainable with higher statistical power. For the three patients with VTT who completed the ICI treatment, two of them exhibited a prominent reduction in the primary tumor mass, but not of the VTT, and we speculated that the VTTs’ lack of response to ICI may be related to the genetic features of VTT. However, we were unable to show this directly, and instead, it was deduced from the genetic features identified in the VTT samples from the group that underwent surgery. The three patients were unfit for surgery, and therefore no tissue was available for genetic testing pre- and post-ICI treatment. Nevertheless, the differing responses to ICI highlight the potential differences in the immune microenvironment between the primary tumor and VTT in ccRCC patients, and this merits further study on additional surgical cases that are also treated with ICI.

## Data Availability Statement

The datasets presented in this study can be found in online repositories. The names of the repository/repositories and accession number(s) can be found below: https://bigd.big.ac.cn/gsa-human/, HRA000795.

## Ethics Statement

The studies involving human participants were reviewed and approved by the ethics committee of the First Medical Center of PLA General Hospital (S2017-100-01). The patients/participants provided their written informed consent to participate in this study.

## Author Contributions

SN: data collection and drafting the article. KL: data analysis and manuscript revision. CP: data collection and analysis. YY: data collection. QH: data collection. SW: data collection. BC: data collection. XM: data collection and design of this work. XZ: design of this work. BW: design of this work and data analysis. All authors contributed to the article and approved the submitted version.

## Funding

This study was supported by the National Natural Science Foundation of China (No. 81970594) and the Outstanding Youth Training Program of PLA General Hospital.

## Conflict of Interest

The authors declare that the research was conducted in the absence of any commercial or financial relationships that could be construed as a potential conflict of interest.
